# Source-bound perceptual persistence in chronic orofacial symptoms: a hypothesis framework

**DOI:** 10.22514/jofph.2026.057

**Published:** 2026-09-12

**Authors:** Paul A.M. Versteegh

**Affiliations:** ^1^Vogellanden Centre of Rehabilitation Medicine & Special Care Dentistry, 8013 XZ Zwolle, The Netherlands

**Keywords:** Orofacial pain, Perceptual persistence, Symptom perception, Occlusal dysesthesia, Persistent dentoalveolar pain, Burning mouth syndrome, Temporomandibular disorder, Nociplastic pain, Source attribution, Active sampling

## Abstract

Persistent orofacial symptoms often remain locally compelling, disabling, and treatment-resistant when their ongoing burden is insufficiently explained by current nociceptive input, neurogenic pathology, or other identifiable peripheral findings. This narrative, theory-driven review develops source-bound perceptual persistence as a hypothesis-generating framework for understanding such conditions across pain-dominant, dysaesthetic, and occlusal-percept presentations. The central construct is source dominance in perceptual persistence: the extent to which a suspected orofacial source continues to organize perception, action, treatment seeking, clinical reasoning, and expectations of recovery. The framework is informed by four partially overlapping literatures concerning signal availability in sensory and pain systems, symptom-perception mechanisms of credibility and failed updating, orofacial sensorimotor mechanisms of active resampling, and clinical embedding mechanisms of source stabilization. We propose that chronic orofacial persistence may arise through a recurrent maintenance loop in which sensory evidence remains available, becomes credible as bodily evidence, is repeatedly resampled through oral action, and becomes clinically or biographically embedded around a suspected orofacial source. The framework helps explain why pain, burning, pressure, dysaesthesia, occlusal wrongness, or other orofacial experiences may differ in sensory quality and in their dominant mechanisms, yet converge when they remain perceptually real, accessible to repeated oral testing, and clinically actionable. Source-bound perceptual persistence is proposed as an integrative maintenance framework for generating empirically testable hypotheses about how chronic orofacial symptoms remain organized around suspected sources when current peripheral findings no longer adequately explain the ongoing symptom burden.

## 1. Introduction

Persistent physical symptoms are increasingly recognized as a major problem across medicine. They refer to distressing somatic complaints that may persist across somatic disease, functional somatic disorders, mental disorders, and undiagnosed conditions [1, 2]. Their clinical burden can remain substantial when current tissue pathology, disease activity, or identifiable pathophysiology no longer sufficiently explains the symptom burden [1]. Such symptoms are best understood as bodily experiences that are clinically real and consequential, while being insufficiently captured by currently available biomedical explanation [3]. Contemporary symptom-perception models similarly emphasize interactions among bodily signals, attention, expectation, interoceptive calibration, affective relevance, and failed updating [4, 5, 6]. Symptom persistence therefore requires models that move beyond a simple organic-versus-psychological distinction.

This problem is especially relevant to chronic orofacial pain and related orofacial symptoms. Temporomandibular disorders (TMD), persistent dentoalveolar pain (PDAP), persistent idiopathic facial pain (PIFP), burning mouth syndrome (BMS), chronic myogenous orofacial pain, selected trigeminal neuropathic pain conditions, and occlusal dysesthesia (OD) remain clinically distinct. Yet they share a recurring challenge: symptoms may remain locally compelling, disabling, and treatment-resistant when their ongoing burden is no longer sufficiently explained by current nociceptive, neurogenic, odontogenic, mucosal, musculoskeletal, occlusal, or other identifiable peripheral findings.

A previous theory-driven comparison of selected persistent orofacial conditions proposed that diagnostically distinct symptom forms can be compared at the level of their maintenance mechanisms once current peripheral findings no longer adequately explain the ongoing burden [7]. That comparison identified shared maintenance domains, including reduced proportionality to current peripheral findings, altered sensory or central processing, attentional and affective salience, behavioural responses, and clinical embedding. The present review builds on that maintenance-domain approach by asking a more specific question: how may such processes become organized around a specific, suspected orofacial source? In this review, source-bound perceptual persistence refers to the continued organization of an orofacial experience around such a suspected source when the ongoing symptom burden is no longer fully explained by current peripheral findings.

Several partially overlapping literatures are relevant to this question. Pain neuroscience explains how sensory or pain-related evidence can remain available through altered sensory gain, peripheral or central sensitization, nociplastic mechanisms, and descending pain modulation [8, 9, 10, 11, 12]. These mechanisms are especially relevant to the present pain-dominant presentations.

Symptom-perception models explain how available bodily signals acquire salience, credibility, and symptom meaning. Symptom perception is an inferential process shaped by prior expectations, attention, context, and interoceptive calibration [4, 5, 13]. Persistent physical symptoms have been conceptualized as perceptual dysregulation, in which expectations and prior models shape the experience itself [5]. Expectation models further suggest that disconfirming information, including medical reassurance, may fail to update the expectation that something remains wrong with the body [6]. This helps explain why symptoms can remain credible and concerning despite repeated normal or non-explanatory clinical findings [1, 4, 5].

The orofacial system adds a distinctive sensorimotor dimension. The mouth, teeth, periodontal tissues, mucosa, masticatory muscles, temporomandibular joints, and trigeminal territories support biting, chewing, swallowing, speaking, oral exploration, taste, smell-related flavour perception, and facial expression [14, 15]. These functions integrate touch, proprioception, temperature, pain, gustation, olfactory flavour components, and motor feedback during ordinary oral behaviour [15]. Taste and smell are clinically relevant, especially in conditions such as burning mouth syndrome, in which dysgeusia, dry-mouth-like sensations, and altered oral sensory experience may coexist. In the present framework, however, the main focus is on those orofacial sources that can remain accessible to repeated testing through oral behaviour or intervention: teeth, occlusion, mucosal surfaces, masticatory muscles, temporomandibular joints, and trigeminal territories.

Dental and occlusal changes can induce plasticity in the facial motor cortex and adjacent somatosensory cortex, indicating that adaptation to altered occlusion or tooth loss is an active brain–body process [16, 17]. In addition, occlusal discomfort and OD may involve prefrontal evaluative or regulatory processes as well as peripheral tooth contact [18, 19, 20]. The suspected orofacial source is therefore often repeatedly accessible to action. This allows active orofacial resampling: repeated biting, clenching, palpation, tongue exploration, jaw movement, clinical testing, imaging, adjustment, splinting, restoration, or other source-directed interventions.

Clinical and psychosomatic models add the domains of attribution, metacognition, body memory, and clinical embedding. Persistent symptoms are increasingly understood as clinically real bodily experiences shaped by interactions among bodily signals, attention, expectation, affective relevance, behaviour, and clinical context [1, 4, 5, 6]. Metacognitive beliefs about monitoring, control, and explanation may maintain symptom-focused attention and threat monitoring [21]. Previous bodily experiences, including painful procedures, altered proprioceptive states, failed treatment, or distressing clinical encounters, may shape current symptom perception and behaviour [22]. These processes are especially relevant in orofacial care, where symptoms often remain organized around specific anatomical sites.

Taken together, these literatures suggest that the key problem is not symptom continuation alone, but continued organization of symptoms around a suspected source that remains salient and credible. Pain is itself a perceptual experience, yet chronic orofacial complaints are not always pain-dominant. Some patients primarily report burning, pressure, dysaesthesia, altered oral bodily state, or a persistent sense that the bite is wrong. These experiences differ in sensory quality and in their dominant mechanisms, but they may converge when a tooth, bite, mucosal surface, masticatory muscle, temporomandibular joint, or trigeminal territory continues to organize perception, action, clinical care, and expectations of recovery.

The central construct is source dominance: the extent to which a suspected orofacial source determines what counts as evidence, what actions follow, what treatments remain conceivable, and what recovery is understood to mean. The proposed novelty lies in explaining how established neurobiological, perceptual, sensorimotor, and clinical processes may converge to produce and sustain this source-bound organization.

The aim of the present review is to develop source-bound perceptual persistence as a hypothesis-generating maintenance framework for chronic orofacial symptoms. The framework complements existing diagnostic and disease-mechanism models by generating testable hypotheses about how source dominance may arise and contribute to symptom persistence.

## 2. Methods

This article presents a narrative, theory-driven review and conceptual synthesis. A narrative approach was chosen because source-bound perceptual persistence is a proposed framework rather than an established diagnosis or standardized outcome amenable to systematic review or meta-analysis. The review integrates relevant literature to generate testable hypotheses about how chronic orofacial symptoms may remain organized around a suspected source.

The present synthesis derives four source-bound process steps from the maintenance domains identified in a previous theory-driven comparison of persistent orofacial conditions [7].

Relevant literature was identified through iterative searches in PubMed, APA PsycNet, and Google Scholar, supplemented by backward and forward citation tracking of key classification papers, reviews, consensus statements, mechanistic studies, and condition-specific empirical papers. Searches were conducted iteratively and were most recently updated in June 2026. Search terms combined chronic orofacial pain, TMD, PDAP, PIFP, BMS, OD, phantom bite syndrome, and related dysaesthetic or occlusal-percept conditions with broader terms for symptom persistence, sensory processing, pain modulation, symptom perception, expectation, interoception, oral sensorimotor behaviour, source attribution, and clinical embedding.

Literature was considered relevant when it defined or classified one of the relevant orofacial conditions, introduced a concept needed to explain source-bound organization, provided direct orofacial mechanistic evidence, or clarified clinical risks of repeated local intervention and persistent source attribution. Literature was excluded when it concerned acute pathology without relevance to persistence, general psychological distress without a symptom-perception or orofacial link, treatment outcomes without mechanistic or conceptual relevance, or peripheral pathology that provided a sufficient explanation for the symptom burden.

During the synthesis, the broader maintenance-domain approach was refined into four source-bound process steps by asking what keeps sensory or pain-related evidence available, what makes it credible as bodily evidence, what repeatedly regenerates it through orofacial action, and what stabilizes the suspected source within clinical and biographical history. These questions organized the synthesis into four domains: signal availability, symptom/percept credibility, active orofacial resampling, and source stabilization. The four steps were used as organizing functions for hypothesis generation, not as established mechanisms or diagnostic criteria.

Foundational papers were retained when they introduced concepts central to the argument, whereas recent reviews and empirical studies were prioritized when they updated, refined, or challenged existing interpretations. References were considered key when they defined a retained condition, introduced a central mechanism, provided direct orofacial evidence for that mechanism, or clarified the limits of applying that mechanism across pain-dominant and non-pain presentations. To reduce the risk of confirmatory selection, literature was also retained when it constrained or challenged the proposed synthesis, including work emphasizing ongoing peripheral pathology, condition-specific mechanisms, the limits of nociplastic pain in non-pain conditions, and critiques of overextended interpretations based on predictive-processing or free-energy interpretations. Neuroimaging, sensory-testing, and blood-flow findings were interpreted as candidate correlates or mechanistic clues rather than diagnostic biomarkers.

## 3. Conceptual synthesis

### 3.1 Organizing framework: a source-bound maintenance loop

Building on the maintenance domains identified in the previous review, the present synthesis organizes the relevant literature into four explanatory process steps: signal availability, symptom/percept credibility, active orofacial resampling, and source stabilization.

The four process steps were derived from four explanatory questions that follow from the source-bound problem addressed in this review. The first question is why sensory or pain-related evidence remains available to perception; this defines signal availability. The second question is why available evidence acquires the status of meaningful bodily evidence rather than being treated as harmless background sensation; this defines symptom/percept credibility. The third question is how a suspected source that is accessible through orofacial action can be repeatedly tested and thereby regenerated as sensory evidence; this defines active orofacial resampling. The fourth question is how attribution, treatment history, body memory, reassurance seeking, and clinical interactions may stabilize the source over time; this defines source stabilization.

The steps are therefore distinguished by the explanatory question from which they are derived. They may overlap in practice because attention, salience, and action-readiness can contribute to each of them. They are therefore not proposed as independent mechanisms with sharp boundaries. However, they are also not simply four descriptions of persistent attention. Persistent attention describes continued focus on a symptom or bodily site. The present framework asks a more specific source-bound question: how available sensory evidence becomes credible, is repeatedly regenerated through orofacial action, and becomes stabilized around a suspected source that remains clinically actionable. The construct that captures this convergence is source dominance. Signal availability, symptom/percept credibility, active orofacial resampling, and source stabilization are therefore treated as supporting process steps that specify how a suspected orofacial source may become and remain dominant. Together, they form a proposed maintenance loop through which the source may continue to organize perception, action, treatment seeking, clinical reasoning, and expectations of recovery.

To reduce ambiguity, Table 1 defines the core terms used in the framework, specifies their role in the proposed maintenance loop, and lists candidate indicators for future empirical work. Terms were retained only when they carried a distinct conceptual or operational function. The four process steps are informed by distinct but converging literatures; their integration into a recurrent source-bound maintenance loop is the central hypothesis advanced by this review. Table 2 (Ref. [4, 5, 8, 9, 11, 12, 13, 23, 24, 25, 26, 27, 28, 29, 30, 31, 32, 33, 34, 35, 36]); Table 3 (Ref. [1, 4, 5, 6, 13, 21, 31, 37, 38, 39, 40, 41, 42, 43, 44, 45, 46, 47, 48]); Table 4 (Ref. [15, 16, 17, 18, 19, 20, 24, 27, 36, 49, 50, 51, 52, 53, 54, 55, 56, 57, 58, 59, 60, 61, 62, 63, 64]); Table 5 (Ref. [1, 3, 5, 6, 19, 20, 21, 22, 24, 31, 39, 40, 45, 47, 52, 62, 63, 64, 65, 66, 67, 68, 69]) summarize the representative literatures informing each step.

**Table 1.** Key terms used in the source-bound perceptual persistence framework.

Term	Role in framework	Working definition	Candidate indicators
Source-bound perceptual persistence	Integrative framework	Continued organization of an orofacial experience around a suspected source when ongoing symptom burden is insufficiently explained by current peripheral findings.	Persistent or recurrent orofacial pain, dysaesthesia, burning, pressure, occlusal wrongness or altered oral bodily state, together with continued source-directed attention, testing, treatment seeking or recovery expectations.
Suspected orofacial source	Descriptive clinical anchor	The tooth, bite, mucosal surface, masticatory muscle, temporomandibular joint, trigeminal territory, or other orofacial site around which the symptom is organized.	Patient-reported source, examination focus, onset narrative, repeated self-testing, treatment history, or recovery expectation directed toward a specific oral site or territory.
Source dominance	Candidate measurable construct	The extent to which a suspected source determines what counts as evidence, what actions follow, what treatments remain conceivable, and what recovery is understood to mean.	Attentional occupation, checking or testing, reassurance seeking, source-directed treatment seeking, avoidance or protection, and recovery expectations focused on correction, removal, relaxation, or normalization of the source.
Signal availability	Process step	Continued availability of sensory or pain-related evidence through residual input, increased gain, impaired inhibition, internally generated input, or inferred sensory evidence.	Persistent or fluctuating pain, dysaesthesia, burning, pressure, wrongness, hypersensitivity, spread, sensory intrusiveness, or recurrence despite insufficient current peripheral explanation.
Symptom/percept credibility	Process step	The process by which available sensory evidence becomes experienced as meaningful bodily evidence of an unresolved or clinically relevant problem.	Perceived bodily certainty, threat value, failed reassurance, difficulty dismissing the sensation, and interpretation of ordinary or fluctuating signals as clinically meaningful.
Active orofacial resampling	Process step	Repeated testing or probing of the suspected source through orofacial action or clinical examination.	Bite checking, clenching, tapping, chewing tests, tongue exploration, swallowing checks, jaw movement testing, palpation, repeated occlusal or dental testing, or requests for renewed examination.
Source stabilization	Process step	The process by which attribution, treatment history, body memory, metacognitive certainty or wrongness, reassurance seeking, and clinical interaction may stabilize the suspected source as clinically actionable.	Persistent attribution to a specific source, symptom meaning organized around a procedure or treatment episode, repeated local interventions, continued correction seeking, ongoing clinical focus on the source, and recovery expectations tied to local correction or normalization.

*Terms are used as heuristic and operational concepts for organizing the reviewed literature and generating empirically testable hypotheses. The role column indicates whether a term functions as an integrative framework, clinical anchor, candidate measurable construct, or process step. Candidate indicators are illustrative and require empirical validation; they are not proposed as diagnostic criteria or established mechanisms.*

**Table 2.** Signal availability: key literatures informing sensory and pain-system mechanisms.

Process step	Contribution to source-bound perceptual persistence	Key references
Triggered or spontaneous signal emergence	Persistent orofacial experiences may follow identifiable dental, mucosal, musculoskeletal, neurogenic, or treatment-related events, but may also arise without an identifiable precipitating trigger.	[23, 24, 25, 26]
Residual or inferred sensory evidence	Sensory evidence may arise from residual oral input but may also be internally generated or inferred when current peripheral support is minimal or clinically insufficient.	[4, 5, 13]
Increased sensory gain	Weak or ordinary oral signals may become more available to perception when somatosensory or nociceptive gain is increased.	[27]
Altered nociceptive processing	Pain may persist through peripheral or central sensitization, altered trigeminal nociceptive excitability, or nociplastic mechanisms when current tissue damage or somatosensory lesions do not sufficiently explain the experience.	[8, 9, 27]
Shared pain-system vulnerability	TMD and other chronic overlapping pain conditions may share broader nociplastic or pain-system vulnerabilities.	[28, 29]
Descending inhibition or facilitation	Cortical and brainstem systems can inhibit or facilitate nociceptive and trigeminal transmission, shaping whether input remains clinically salient.	[11, 12, 30]
Failure of backgrounding	Oral signals that are normally predictable, self-generated, and relatively low in attentional priority may remain foregrounded when they are weighted as informative or action-relevant.	[4, 31]
Affective-motivational amplification	Affective, motivational, and learning systems may increase the urgency, behavioural relevance, and persistence of pain or bodily experience.	[32, 33]
Pain as behavioural control signal	Pain may function as a behavioural control signal by guiding avoidance, protection, decision-making, and attempts to control or correct the perceived source.	[34, 35]
Mixed orofacial pain mechanisms	Chronic orofacial pain may involve mixed nociceptive, neuropathic, nociplastic, modulatory, and network mechanisms rather than a single current local mechanism sufficient to account for the symptom burden.	[25, 27, 36]

*This table summarizes literature relevant to the first process step: how bodily or pain-related signals may remain available to perception despite insufficient current peripheral support, when the symptom burden is no longer adequately explained by current peripheral findings. References are representative rather than exhaustive and were selected to identify the main explanatory literatures informing each process step. TMD: temporomandibular disorders.*

**Table 3.** Symptom/percept credibility: key literatures informing percept-system mechanisms.

Process step	Contribution to source-bound perceptual persistence	Key references
Selection and filtering of bodily signals	Available bodily signals are filtered, attended to, amplified, and interpreted before being experienced as symptom.	[1, 4, 5]
Inferential symptom formation	Symptom perception depends on afferent input, expectations, context, and precision weighting rather than passive readout of peripheral signals.	[4, 5]
Interoceptive prediction and regulation	Bodily experience is shaped by predictions about body state and processes involved in allostatic regulation.	[13, 37]
Salience and threat assignment	Bodily signals become foregrounded when they are behaviourally relevant, threatening, uncertain, or difficult to ignore; this may involve distributed interoceptive, salience, prefrontal, and pain-modulatory networks.	[4, 5, 6, 13, 38]
Illness representation	The percept is organized into an illness model involving identity, cause, timeline, consequences, controllability, and treatment expectations.	[39]
Failed updating by reassurance	Normal findings or reassurance may fail to revise the expectation that something remains wrong.	[4, 6]
Metacognitive confidence or wrongness	Feelings of certainty, uncertainty, familiarity, or wrongness may make the percept credible and action-guiding.	[21, 40, 41]
Cognitive-affective vulnerability	Depression, loneliness, alexithymia, catastrophizing, attention deficits, or executive difficulties may be associated with greater symptom burden and increased monitoring.	[42, 43, 44, 45]
Reassurance seeking and checking	Repeated reassurance or checking may temporarily reduce uncertainty while maintaining threat-focused monitoring.	[6, 21]
Orofacial perceptual modulation	Occlusal tactile perception varies with anxiety and catastrophizing, indicating potential central modulation of oral perceptual experience.	[46]
Conceptual caution	Predictive-processing terminology is useful for expectation, precision, and updating, but should remain heuristic.	[31, 47, 48]
Uncertainty and unresolvedness	Uncertainty, threat, failed reassurance, loss of control, or prior treatment experience may make available sensory evidence more credible as evidence of an unresolved bodily problem.	[5, 6, 21]

*This table summarizes literature relevant to the second process step: how available bodily signals may acquire salience, credibility, symptom meaning, and resistance to updating. References are representative rather than exhaustive and were selected to identify the main explanatory literatures informing each process step.*

**Table 4.** Active orofacial resampling: key literatures informing orofacial sensorimotor mechanisms.

Process step	Contribution to source-bound perceptual persistence	Key references
Precise oral somatosensory input	Periodontal mechanoreceptors, oral tactile acuity, and trigeminal proprioception provide precise input for tooth contact, force control, and jaw function.	[49, 50, 51]
Occlusion as sensorimotor adaptation	Occlusion depends on central modulation of periodontal, dental, and mucosal input and on sensorimotor neuroplasticity, rather than on mechanical factors alone.	[52]
Plastic oral body schema	Perceived mouth and tooth position can be recalibrated by multisensory experience.	[53, 54]
Cortical adaptation to oral change	Dental or occlusal change, tooth loss, and altered oral input can induce plasticity in the facial motor cortex and adjacent somatosensory cortex.	[16, 17, 55]
Multisensory oral sensorimotor control	Biting, chewing, swallowing, speech, and oral exploration depend on integrated tactile, proprioceptive, gustatory, nociceptive, thermal, and motor information rather than on isolated peripheral input.	[15, 56, 57]
Active resampling of the suspected source	Biting, chewing, clenching, tapping, tongue exploration, swallowing, and clinical testing can repeatedly regenerate sensory evidence from the suspected source.	[52, 57]
Failed attenuation of self-generated sensation	Self-generated oral sensations should normally be attenuated; reduced attenuation may keep bite, tongue, tooth, or mucosal sensations intrusive.	[58, 59]
Experimental occlusal discomfort and prefrontal monitoring	Experimentally induced occlusal discomfort engages prefrontal hemodynamic responses, suggesting evaluative or regulatory cortical involvement in occlusal perception.	[19, 60]
OD/PBS central regulation during oral function	OD/PBS findings implicate altered prefrontal activation, chewing-related regulation, and cerebral blood-flow patterns in persistent bite-focused complaints.	[18, 20, 61]
Maladaptive occlusal checking	OD illustrates persistent bite-focused perception, checking, and correction-seeking despite insufficient objective occlusal explanation.	[52, 62]
Pain–sensorimotor coupling	Pain and sensorimotor behaviour reciprocally influence each other, shaping jaw use, guarding, avoidance, and symptom sampling.	[57]
Source-bound clinical examples	PDAP, BMS, and chronic TMD illustrate tooth-region, oral-state, and jaw-function patterns in which symptoms may remain linked to specific sources despite adaptation or treatment.	[24, 27, 36, 63, 64]

*This table summarizes literature relevant to the third process step: how oral action may repeatedly resample the suspected source and regenerate sensory evidence. References are representative rather than exhaustive and were selected to mark the main explanatory literatures informing each process step. OD: occlusal dysesthesia; PDAP: persistent dentoalveolar pain; BMS: burning mouth syndrome; TMD: temporomandibular disorders; PBS: phantom bite syndrome.*

**Table 5.** Source stabilization: key literatures informing clinical embedding mechanisms.

Process step	Contribution to source-bound perceptual persistence	Key references
Clinical and social predicament	Symptoms insufficiently explained by current biomedical findings remain real and clinically consequential rather than being dismissed as “psychogenic”.	[1, 3, 65]
Attribution to an orofacial source	The experience becomes organized around a concrete oral object or territory, such as a tooth, bite, mucosal site, muscle, joint, or trigeminal region.	[39]
Illness narrative and treatment meaning	The symptom is embedded in narratives about cause, onset, timeline, consequences, controllability, and treatment response.	[39]
Treatment history and body memory	Prior dental procedures, pain episodes, emotional experiences, or distressing clinical events may shape current body representation and symptom meaning.	[22, 66]
Metacognitive stabilization	Feelings of certainty, uncertainty, familiarity, wrongness, or unresolvedness may stabilize monitoring and treatment seeking.	[21, 40]
Repeated local intervention	Interventions may reinforce attribution to the suspected source when current peripheral findings are insufficient to explain the ongoing symptom burden.	[62, 63, 64]
Bite-focused clinical reinforcement	Repeated occlusal adjustment or restorative correction may maintain the bite as the central explanatory object.	[52, 62]
OD as clinically embedded wrongness	OD illustrates a persistent, clinically actionable sense of bite abnormality despite insufficient objective occlusal explanation.	[19, 20, 62]
PDAP as clinically embedded tooth-region pain	PDAP may remain experienced as localized tooth-region pain and may be associated with repeated dental assessment or intervention.	[63, 64]
BMS, stigma, and invalidation	BMS may involve diagnostic uncertainty, stigma, dismissal, or perceived discrimination, which may contribute to symptom burden and intensify efforts to obtain an explanation.	[24, 67]
Embodied and social disruption	Persistent symptoms may disrupt embodied engagement, bodily trust, self-regulation, and participation; improvement may involve restoring confidence in embodied function.	[5, 45, 68]
Conceptual safeguards	Predictive-processing and FEP terminology should remain heuristic and hypothesis-generating, not universal or reified.	[31, 47, 69]
Reassurance seeking and checking	Repeated reassurance or checking may temporarily reduce uncertainty but can maintain threat-focused monitoring by making the symptom or source the object of renewed evaluation.	[6, 21]

*This table summarizes literature relevant to the fourth process step: how attribution, treatment history, body memory, metacognitive monitoring, and clinical interaction may stabilize the suspected source as a clinically actionable problem. References are representative rather than exhaustive and were selected to mark the main explanatory literatures informing each process step. OD: occlusal dysesthesia; PDAP: persistent dentoalveolar pain; BMS: burning mouth syndrome; FEP: free energy principle.*

### 3.2 Signal availability: pain-system mechanisms

Table 2 summarizes representative literature informing the first process step: signal availability. This step asks how an orofacial experience remains available to perception when current peripheral findings no longer adequately explain its intensity, duration, spread, recurrence, or clinical impact. Signal availability may follow an identifiable trigger, but it may also arise without a clear precipitating event. In this framework, ongoing tissue damage, active inflammation, dental disease, or a clear nerve lesion represent possible routes by which sensory evidence remains available, but are not the only routes. Sensory evidence may also arise through residual oral input, increased somatosensory or nociceptive gain, altered descending modulation, impaired inhibition, internally generated input, or inferred sensory evidence when current peripheral support is minimal. In pain-dominant presentations, signal availability may further be supported by peripheral and central sensitization mechanisms, including activity-dependent increases in trigeminal nociceptive excitability.

Many oral signals are continuously present during ordinary function yet normally remain in the background because they are predictable, self-generated, and clinically irrelevant. The key question is why some signals remain perceptually available. A predictive-processing vocabulary may describe one part of this process: small oral differences may be assigned increased precision or salience, making them more likely to be treated as informative. This vocabulary is used here heuristically to describe signal weighting, uncertainty, salience, and action-readiness.

Signal weighting also depends on context. Minor tooth contact, mucosal sensation, pressure, muscular input, joint sensation, or trigeminal input may remain foregrounded when uncertainty, threat, previous treatment failure, failed reassurance, or loss of confidence makes such input clinically meaningful. Under these conditions, the decisive factor is not only the magnitude of the signal, but also the state of the perceptual system receiving and interpreting it.

Signal availability therefore helps explain why an experience can continue to recur, fluctuate, or remain intrusive. It is a necessary but incomplete step in the proposed loop. Available sensory or pain-related evidence must also become credible as bodily evidence and remain linked to a suspected orofacial source. That transition is addressed in the next section.

### 3.3 Symptom/percept credibility: percept-system mechanisms

Table 3 summarizes representative literature informing the second process step: symptom/percept credibility. This step asks how available sensory evidence becomes experienced as meaningful bodily evidence of an unresolved or clinically relevant problem.

The key transition is from sensory evidence to bodily evidence. A weak or ambiguous sensation may become clinically important when it is interpreted as evidence that a bodily source is abnormal or unresolved.

This transition is especially likely under conditions of uncertainty and unresolvedness. Such concern may arise when the patient cannot determine whether the sensation is harmless or pathological, when the sensation is interpreted as indicating possible damage or loss of function, when previous treatment has failed, when explanations conflict, or when reassurance leaves the bodily experience unchanged. Under these conditions, the perceptual system may treat the sensation as information about the state of the body.

Psychological and social factors enter this step by altering the perceived credibility and significance of sensory evidence. Anxiety, catastrophizing, depressive symptoms, loneliness, invalidation, loss of trust, or previous negative clinical encounters may increase vigilance, threat appraisal, and the need for explanation. They may therefore make an ambiguous oral signal more likely to be experienced as salient, credible, and difficult to dismiss. At the neural level, salience, monitoring, and action-readiness are likely to involve distributed interoceptive, prefrontal, and pain-modulatory networks. In the present framework, these networks are treated as candidate correlates of symptom/percept credibility rather than diagnostic biomarkers of source-bound persistence.

This may help explain why reassurance may fail. The clinician may be responding to negative findings, while the patient is responding to persistent bodily evidence. A normal radiograph, acceptable occlusion, or normal mucosal examination may reduce clinical suspicion, while the symptom can remain perceptually credible if the source continues to feel wrong.

### 3.4 Active orofacial resampling: orofacial sensorimotor mechanisms

Table 4 summarizes representative literature informing the third process step: active orofacial resampling. This step asks why a credible orofacial symptom is repeatedly tested through oral action and how such testing may regenerate sensory evidence from the suspected source.

The orofacial region is clinically important because many suspected sources are continuously accessible to action. Chewing, swallowing, clenching, speaking, jaw positioning, tongue exploration, and clinical testing can all become ways of evaluating whether the suspected source continues to feel abnormal.

Orofacial action does more than passively observe the suspected source; it changes the sensory state being evaluated. Oral functions depend on integrated tactile, proprioceptive, nociceptive, motor, gustatory, thermal, and affective feedback. Occlusion and jaw function also depend on sensorimotor adaptation and central modulation of periodontal, dental, and mucosal input. Repeated source-directed action may therefore regenerate an integrated sensory state in which contact, pressure, proprioception, discomfort, salience, and expectation are combined.

Source-directed testing is therefore clinically meaningful. Each orofacial action or clinical examination can produce additional sensory and perceptual information associated with the same source. When that source is already credible as the origin of the problem, new input may strengthen source attribution, increase monitoring, or renew the perceived need for explanation or correction. The patient is therefore not only thinking about the symptom or remembering it; the oral system repeatedly provides opportunities to resample the suspected source as a sensory and motor event.

This mechanism may be adaptive when ongoing pathology, injury, inflammation, overload, or functional limitation requires monitoring or protection. It becomes part of source-bound persistence when repeated sampling continues after the current peripheral explanation has become insufficient. In that situation, active orofacial behaviour may help maintain the symptom as perceptually real and continue to link it to the suspected source.

### 3.5 Source stabilization: clinical embedding mechanisms

Table 5 summarizes representative literature informing the fourth process step: source stabilization. This step asks how a repeatedly sampled orofacial experience becomes stabilized as a problem attributed to a specific oral source. Repeated sensory and clinical experiences occur within a clinical history. A dental procedure, extraction, occlusal adjustment, painful episode, failed treatment, invalidating consultation, or unresolved diagnosis can become the temporal anchor for the symptom. Subsequent sensations are then interpreted in relation to that history. The source becomes both a current sensory location and a clinically meaningful site of bodily concern.

This helps explain why a symptom may remain organized around a specific source. Source stabilization is supported by attribution, treatment history, body memory, metacognitive feelings of certainty or wrongness, and repeated attempts to obtain an explanation or correction. These processes shape how later sensations are noticed, interpreted, tested, and acted upon.

At this stage, local treatment may sometimes contribute to maintaining the loop. When no correctable local abnormality adequately explains the symptom burden, further procedures may keep the suspected source clinically active and repeatedly testable.

### 3.6 Integrated synthesis: from maintenance loop to source dominance

The four process steps describe interacting contributions to source-bound perceptual persistence. Their proposed integration forms a recurrent maintenance loop. The loop may continue after the effects of the local, neurogenic, or treatment-related trigger no longer adequately explain the current symptom burden. This clarifies what persists: not only pain, discomfort, burning, pressure, dysaesthesia, or occlusal awareness, but the organization of experience around a source.

Different orofacial conditions may enter the loop through different dominant pathways. Pain-dominant, dysaesthetic, and occlusal-percept presentations may differ in initiating mechanisms and sensory quality yet converge when a suspected orofacial source remains perceptually real and continues to organize recovery expectations.

Source dominance is the emergent organizing construct proposed by this framework. The four process steps specify how it may arise, be maintained, and feed back into attention, resampling, and treatment seeking.

Fig. 1 summarizes the proposed source-bound maintenance loop.

**Fig. 1.** Proposed source-bound maintenance loop. The four process steps interact around a suspected source and may give rise to source dominance. TMJ: temporomandibular joint.

## 4. Discussion

### 4.1 Main implication: persistence as a source-bound maintenance loop

The central implication of this review is that chronic orofacial persistence may be conceptualized as source dominance operating within a source-bound maintenance loop. The relevant shift is from symptom presence alone to the way a symptom remains organized around a suspected source.

The framework integrates mechanisms described in predictive-processing, symptom-perception, nociplastic pain, pain-modulation, illness-representation, metacognitive, and clinical-embedding literatures. Its contribution lies in specifying how these mechanisms may converge in the orofacial situation, where a suspected source can remain concrete, accessible to repeated oral testing, and available for clinical intervention. A suspected orofacial source can therefore remain dominant even when the symptom burden is no longer adequately explained by current peripheral findings.

This source-bound organization gives persistent orofacial symptoms their clinical force. The experience remains perceptually real because it continues to be experienced as arising from a specific site. It remains accessible to repeated testing because the patient can repeatedly reproduce or probe it through orofacial actions. It remains clinically relevant because the suspected source can be examined, explained, protected, adjusted, restored, extracted, splinted, medicated, imaged, or otherwise treated.

Persistence therefore involves more than recurrence of pain, burning, pressure, discomfort, dysaesthesia, or occlusal awareness. It involves source dominance: the suspected orofacial source may influence what counts as evidence, what actions follow, what treatments remain conceivable, and what recovery is allowed to mean. This formulation preserves the bodily reality of the symptom while directing attention to the loop through which the source remains dominant across perception, behaviour, and care.

### 4.2 Operationalizing source dominance

Source dominance extends beyond localization or source attribution. Many acute and chronic symptoms are experienced as arising from a specific bodily site, and such source orientation is often adaptive because it can remain responsive to changes in the underlying condition. In source-bound perceptual persistence, by contrast, the suspected source continues to organize attention, behaviour, clinical action, and recovery expectations even when current findings no longer adequately explain the ongoing symptom burden.

Source dominance can be operationalized through observable indicators, although no validated scale for this construct currently exists. Candidate indicators include the time and attention occupied by the source; interpretation of ordinary or fluctuating sensations as evidence that the source remains abnormal; source-directed checking, repeated self-testing, protective behaviour, or avoidance of oral function; reassurance seeking; and continued pursuit of further assessment or correction despite insufficient explanatory findings.

Recovery expectations are especially informative. Source dominance would be expected to be greater when improvement is conceivable mainly as correction, removal, relaxation, or normalization of the source. It would be lower when improvement can also be understood as reduced monitoring, reduced checking, improved oral function, reduced threat, less interference, diminished treatment seeking, or restored confidence in oral function despite residual sensation.

Future studies could examine whether higher source dominance is associated with symptom persistence, disability, repeated treatment seeking, reduced response to reassurance, or poor outcome after further local intervention. Longitudinal or intervention studies could also test whether reductions in checking, threat interpretation, correction-focused recovery expectations, or source-related attentional occupation are associated with reduced source dominance even when residual sensation remains. Source dominance is therefore proposed as a candidate construct for measurement and hypothesis testing.

### 4.3 Perceptual source organization

In pain-dominant conditions, including painful TMD, PDAP, PIFP, BMS, chronic myogenous orofacial pain, and selected trigeminal neuropathic pain conditions, pain-system mechanisms may provide much of the sensory intensity, urgency, and disability [10, 27, 70]. Yet these experiences are also source-organized: pain is experienced as belonging to a tooth, socket, mucosa, muscle, joint, or trigeminal territory [27, 71]. In percept-dominant conditions such as OD, the dominant experience may be wrongness, salience, certainty, dysaesthesia, or the sense that a local source requires correction, with pain intensity playing a smaller role [20, 60].

These formats may overlap in clinical practice. Pain, dysaesthesia, burning, pressure, occlusal wrongness, and related orofacial experiences are all forms of perceptual experience. They differ in phenomenological quality and dominant mechanisms, while all may become organized around a suspected orofacial source.

### 4.4 Why the orofacial region matters

The orofacial region provides a particularly clear setting for source-bound perceptual persistence because it combines multisensory integration, continuous sensorimotor sampling, and concrete clinical modifiability within a small and highly salient functional space. Symptoms in other bodily regions, including the spine, hip, knee, shoulder, or foot, may also become mechanically provoked, protected, investigated, and treated. The mouth makes this process especially visible because suspected sources can be sensed, tested, and modified through everyday oral function and dental or orofacial care.

Oral experience is intrinsically multisensory. Touch, proprioception, taste, temperature, pain, smell-related flavour perception, and motor feedback are integrated during eating, chewing, swallowing, speaking, clenching, and oral exploration. Neuroimaging findings suggest that oral multisensory processing involves crossmodal interactions in which somatosensory and motor regions are frequently implicated [15]. The orofacial sensorimotor system also includes teeth, mucosa, facial skin, masticatory muscles, temporomandibular joints, trigeminal pathways, and central circuits involved in pain, touch, taste, chewing, facial expression, speech, and swallowing [57]. Thus, a suspected source such as a tooth, bite, mucosal surface, muscle, joint, or trigeminal territory is more than a local anatomical site; it can function as a multisensory and sensorimotor perceptual object.

Taste and smell deserve specific mention because they contribute to oral and flavour experience. Taste-related symptoms are clinically relevant in BMS, where burning or dysaesthetic oral sensations may coexist with dysgeusia and xerostomia, although objective taste perception and saliva composition may be normal in some patients [72, 73, 74]. In the present framework, however, the main focus is on those orofacial sources that can remain repeatedly testable through oral behaviour or intervention: teeth, occlusion, mucosal surfaces, masticatory muscles, temporomandibular joints, and trigeminal territories. Chemosensory symptoms may increase oral salience and symptom burden, but, within the present framework, they are less directly accessible to repeated mechanical testing or source-directed local correction than these source-bound sensorimotor anchors.

This has direct relevance for persistence. When a patient checks a bite, explores a mucosal surface with the tongue, clenches, chews, or repeatedly tests jaw movement, the action may regenerate an integrated oral state involving contact, pressure, proprioception, motor feedback, affective salience, and expectation. This interpretation is consistent with evidence that oral functions are fundamentally multisensory, and that orofacial pain and sensorimotor behaviours reciprocally influence one another [15, 57]. The mouth may therefore make source-bound maintenance loops especially visible: the suspected source is continuously sensed, repeatedly self-tested, and often technically available for clinical correction.

### 4.5 Clinical meaning and psychological processes

Psychological and social processes influence how bodily evidence is weighted, interpreted, monitored, and acted upon [1, 5, 6]. Their clinical importance lies in how they may influence source dominance: whether the suspected source remains credible, urgent, and clinically relevant. Uncertainty, threat, previous treatment failure, invalidation, loss of control, catastrophizing, or failed reassurance may increase the perceived credibility and urgency of ambiguous or fluctuating sensations [21, 46]. A weak or ordinary signal may thereby come to feel like evidence that something remains wrong with a specific orofacial source.

This suggests a specific clinical role for psychological and behavioural intervention: reducing the processes that keep the suspected source dominant. Clinically, this may involve addressing uncertainty, reducing checking and reassurance loops, restoring confidence in oral function, and helping the patient tolerate residual or ambiguous sensations while weakening their interpretation as evidence of damage, failed adaptation, or failed treatment.

Existing intervention evidence should be interpreted cautiously. For persistent physical symptoms and bodily distress, psychological interventions appear to have small to moderate effects on symptom severity. Still, the evidence is heterogeneous and does not identify a single preferred technique or a source-dominance-specific treatment [75, 76]. In persistent orofacial pain, psychological care is best framed as multidisciplinary, formulation-guided management aimed at reducing disability, improving function, and modifying maladaptive responses to symptoms [77].

Within the present framework, interventions may reduce source dominance only insofar as they modify the maintaining processes that keep the suspected source credible. Depending on the individual formulation, treatment may target threat and uncertainty, catastrophic interpretation, reassurance seeking, correction-focused expectations, avoidance of oral function, clenching or guarding, repeated oral checking, trauma-related activation, emotionally charged symptom-related memories, or loss of confidence in normal oral use. The specific intervention may be cognitive-behavioural, acceptance-based, mindfulness-based, exposure-based, trauma-focused, relaxation-based, sensorimotor, body-oriented, or habit-focused, but its relevance depends on the maintaining process it addresses rather than on the treatment label itself. These implications are process-based hypotheses derived from the present framework and from formulation-guided approaches to persistent physical symptoms and persistent orofacial pain, rather than established source-dominance treatments [75, 76, 77].

The model also supports caution with repeated local intervention, including occlusal adjustment or restorative correction, when current peripheral findings no longer adequately explain the symptom burden. Local treatment remains appropriate when these findings sufficiently explain the burden. Otherwise, repeated occlusal or dental procedures could potentially reinforce source dominance by keeping the suspected source clinically active and by making recovery depend on further correction [62, 63]. In this situation, dental or occlusal care may contribute more usefully by establishing diagnostic stability, providing a non-blaming explanation, limiting repeated corrective procedures, and supporting confidence in oral function.

### 4.6 Therapeutic implications: source dominance as a candidate treatment target

The practical value of the framework lies in shifting attention from source certainty to source dominance. In many patients with chronic orofacial symptoms, the suspected source is already clear and strongly established. The therapeutic question is whether that source continues to govern attention, checking, treatment seeking, clinical decision-making, and recovery expectations.

When current peripheral findings are insufficient, care may be less effective if it remains organized around increasingly precise attempts to correct, disprove, or reassure patients about the suspected source. A more plausible therapeutic target may be the reduction of source dominance: weakening the extent to which the source controls monitoring, oral behaviour, treatment seeking, and the patient’s expectations of possible recovery. This suggests complementary therapeutic roles. The somatic clinician may establish diagnostic stability and explain source-bound perceptual persistence in a way that validates the patient’s experience. Psychological or behavioural treatment may become relevant when it targets the processes maintaining source dominance.

### 4.7 Limitations and conceptual safeguards 

As a hypothesis-generating framework, source-bound perceptual persistence requires careful interpretation and has important limitations. The present framework is based on a theory-driven narrative review and conceptual synthesis. The literature was selected for conceptual and mechanistic relevance to the proposed process steps. The review provides no exhaustive coverage, formal risk-of-bias assessment, meta-analysis, or graded evidence for each claim. The proposed loop should therefore be read as an integrative hypothesis generated from converging literatures, with empirical validation required in future studies.

Several conceptual boundaries follow from this scope. Nociplastic pain is used as a concept mainly relevant to pain-dominant presentations, especially where altered nociceptive processing, hypersensitivity, or impaired modulation are prominent. Its relevance to percept-dominant conditions such as OD is more limited, because persistence in OD concerns an abnormal occlusal percept rather than pain amplification. Similarly, condition-specific vulnerability factors, including sex-related prevalence patterns, hormonal status, sleep disturbance, and systemic comorbidity, were not developed as separate domains, although they may influence pain-system sensitivity and symptom burden in specific conditions. This is especially relevant to BMS, where neuropathic, mucosal, systemic, hormonal, and psychosocial factors may contribute to symptom burden in ways not fully captured by the present source-bound framework.

The framework applies to cases in which ongoing symptom burden exceeds what current peripheral findings can explain. Appropriate diagnosis and treatment remain essential when active nociceptive, neurogenic, inflammatory, musculoskeletal, dental, mucosal, or structural pathology is present. Source-bound perceptual persistence is therefore best understood as a brain–body–clinical maintenance model for situations in which the current local explanation has become insufficient.

Psychological and social factors are included because they may influence how bodily sensations are interpreted, monitored, and acted upon, and thereby contribute to source dominance. They do not replace nociceptive, neuropathic, sensorimotor, or other bodily mechanisms.

Finally, predictive-processing and related terms are used cautiously. They provide useful vocabulary for expectation, salience, precision, updating, attenuation, and active sampling, but these terms are treated as heuristic tools rather than as a complete ontology of symptom persistence [31, 47, 48]. The value of the present framework lies in whether it generates empirically testable clinical and mechanistic hypotheses about how source-bound perceptual experiences persist, and how their dominance might be reduced.

Because this was a theory-driven narrative review, the possibility of confirmatory selection cannot be fully excluded. This risk was addressed by including literature that constrained the framework, differentiated mechanisms across conditions, or cautioned against overextended explanatory claims.

## 5. Conclusion

This review proposes source-bound perceptual persistence as an integrative hypothesis-generating framework for chronic orofacial symptoms whose ongoing burden is not adequately explained by localized pathophysiological findings. The central innovation of the framework is the construct of source dominance in perceptual persistence: the extent to which a suspected orofacial source continues to organize perception, action, clinical reasoning, treatment seeking, and recovery expectations over time. The four process steps specify how source dominance may emerge and be maintained within a recurrent loop.

This framework can be applied across pain-dominant, dysaesthetic, and occlusal-percept presentations while preserving diagnostic specificity. Painful TMD, PDAP, PIFP, BMS, selected trigeminal neuropathic pain conditions, chronic myogenous orofacial pain, and OD may differ in initiating mechanisms and sensory quality, yet converge when a suspected source remains dominant in perception, action, treatment seeking, and recovery expectations. Source-bound perceptual persistence is therefore proposed as a hypothesis-generating framework for studying how chronic orofacial symptoms remain organized, and how source dominance might be reduced.
